# Electrocardiographic findings associated with early clinical deterioration in acute pulmonary embolism

**DOI:** 10.1111/acem.14554

**Published:** 2022-07-20

**Authors:** Anthony J. Weekes, Jaron D. Raper, Alyssa M. Thomas, Kathryn Lupez, Carly A. Cox, Dasia Esener, Jeremy S. Boyd, Jason T. Nomura, Jillian Davison, Patrick M. Ockerse, Stephen Leech, Eric Abrams, Christopher Kelly, Nathaniel S. O'Connell

**Affiliations:** ^1^ Department of Emergency Medicine Atrium Health's Carolinas Medical Center (Carolinas Medical Center is the Central Site of the Pulmonary Embolism Short‐term Outcomes Registry (PESCOR) consortium) Charlotte North Carolina USA; ^2^ Department of Emergency Medicine Kaiser Permanente San Diego California USA; ^3^ Department of Emergency Medicine Vanderbilt University Medical Center Nashville Tennessee USA; ^4^ Department of Emergency Medicine Christiana Care Newark Delaware USA; ^5^ Department of Emergency Medicine Orlando Health Orlando Florida USA; ^6^ Division of Emergency Medicine University of Utah Health Salt Lake City Utah USA; ^7^ Department of Biostatistics and Data Science Wake Forest School of Medicine Winston‐Salem North Carolina USA; ^8^ Jaron D. Raper, Department of Emergency Medicine University of Alabama at Birmingham Birmingham Alabama USA; ^9^ Alyssa M. Thomas, Emergency Department Houston Methodist Baytown Hospital Houston Texas USA; ^10^ Kathryn Lupez, Department of Emergency Medicine Tufts Medical Center Boston Massachusetts USA; ^11^ Carly A. Cox, Emergency Medicine of Idaho Meridian Idaho USA

## Abstract

**Objectives:**

We sought to determine associations of early electrocardiogram (ECG) patterns with clinical deterioration (CD) within 5 days and with RV abnormality (abnlRV) by echocardiography in pulmonary embolism (PE).

**Methods:**

In this prospective, multicenter study of newly confirmed PE patients, early echocardiography and initial ECG were examined. Initial ECG patterns included lead‐specific ST‐segment elevation (STE) or depression (STD), T‐wave inversion (TWI), supraventricular tachycardia (SVT), sinus tachycardia, and right bundle branch block as complete (cRBBB) or incomplete (iRBBB). We defined CD as respiratory failure, hypotension, dysrhythmia, cardiac arrest, escalated PE intervention, or death within 5 days. We calculated odds ratios (ORs) for CD and abnlRV with univariate and full multivariate models in the presence of other variables.

**Results:**

Of 1676 patients, 1629 (97.2%) had both ECG and GDE; 415/1676 (24.7%) had CD, and 529/1629 (32.4%) had abnlRV. AbnlRV had an OR for CD of 4.25 (3.35, 5.38). By univariable analysis, the absence of abnormal ECG patterns had OR for CD and abnlRV of 0.34 (0.26, 0.44; *p* < 0.001) and 0.24 (0.18, 0.31; *p* < 0.001), respectively. By multivariable analyses, one ECG pattern had a significant OR for CD: SVT 2.87 (1.66, 5.00). Significant ORS for abnlRV were: TWI V_2–4_ 4.0 (2.64, 6.12), iRBBB 2.63 (1.59, 4.38), STE aVR 2.42 (1.58, 3.74), S1‐Q3‐T3 2.42 (1.70, 3.47), and sinus tachycardia 1.68 (1.14, 2.49).

**Conclusions:**

SVT was an independent predictor of CD. TWI V_2–4_, iRBBB, STE aVR, sinus tachycardia, and S1‐Q3‐T3 were independent predictors of abnlRV. Finding one or more of these ECG patterns may increase considerations for performance of echocardiography to look for RV abnormalities and, if present, inform concerns for early clinical deterioration.

## INTRODUCTION

The electrocardiogram (ECG) is one of the first tests performed in the emergency department (ED) on patients with symptoms of chest discomfort or dyspnea. Abnormal ECG findings can be found in cardiopulmonary conditions outside of acute coronary syndrome. Although no specific ECG pattern is diagnostic of acute PE, abrupt pulmonary arterial occlusion may provoke abnormalities of the right ventricle (RV) due to increased pressure, dilatation, or myocardial injury. Abnormal ECG patterns may also occur due to left ventricular (LV) ischemia from combinations of hypoxia or hypotension (decreased oxygen supply) and due to tachycardia (increased oxygen demand). Abnormal ECG patterns associated with RV abnormality (abnlRV) in PE include rightward axis shifts, repolarization abnormalities, ST‐segment myocardial injury patterns, conduction delays, and rhythm disturbances.^1^, ^2^

There is a lack of agreement in the literature on ECG changes in PE and their relation to abnlRV, and one study found no relationship whatsoever.^3^ Prior research in this area has been limited due to retrospective analyses, heterogeneous cohorts of PE patients, small sample sizes, and ECGs not immediately performed at time of PE presentation.^4^, ^5^, ^6^, ^7^, ^8^, ^9^, ^10^, ^11^, ^12^ Few reports involve immediate and contemporaneous evaluations of ECG and echocardiography at PE presentation. Still fewer focus on clinical outcomes that require hospital‐based monitoring or support.

The primary objective of this study was to prospectively determine differences in proportions of predefined ECG patterns between ED patients with PE who experience early clinical deterioration and those who do not. The null hypothesis was there is no difference in proportions of ECG patterns between these two groups of patients. Our secondary objective was to determine the proportions of specific ECG patterns in PE patients with and without abnlRV by goal‐directed echocardiography (GDE). The secondary null hypothesis was there is no difference in the proportions of ECG patterns between those with and without abnlRV. For exploratory objectives, we evaluated (1) the association of mean number of ECG patterns with classifications by PE triaging strategies, and (2) the association of specific ECG patterns with abnormal heart features (LV dysfunction, abnlRV by computed tomography [CT], and elevated biomarkers).

## METHODS

### Study design and setting

This study was a preplanned analysis from a previously reported registry database of ED patients with confirmed acute PE (clinicaltrials.gov NCT02883491 and NCT03915925).^13^ All components of this registry were collected at six urban, academic EDs with emergency medicine residency and advanced emergency ultrasound fellowship programs. A central institutional review board (IRB) approved this federally funded multi‐site study.

### Study population

Adult ED patients (18 years or older) with image confirmed acute PE diagnosed within 12 hours of ED presentation were eligible for enrollment. PE was confirmed by the presence of filling defects in the pulmonary arteries with contrast‐enhanced chest CT or detection of high probability on nuclear ventilation perfusion scan. We excluded patients who refused consent for clinical follow‐up and those who had a ventricular paced rhythm or poor quality ECG tracings that were considered uninterpretable.

### Study protocol

As previously reported, the primary goal of the PE registry was to include early imaging, ECGs, and cardiac biomarkers of ED patients with confirmed PE in the database and determine their association with clinical deterioration.^13^ Research associates at each study site maintain awareness of all ED patients with positive PE studies. In addition, physicians caring for these patients routinely notified the research team and performed GDE. Clinical characteristics and demographic information were collected on each patient. ECGs were performed during the index PE ED visit as part of routine care. Emergency physicians performed GDE during the index PE ED visit, which was subsequently interpreted by faculty of the emergency ultrasound program. ECG and echocardiography were performed independently of each other. The clinical course of patients was monitored by thorough review of the electronic medical record (EMR) or direct communication with inpatient health care providers.

The 12‐lead ECG was completed during the ED management phase. ECGs were performed at the discretion of the treating health care team in response to the patient's symptoms, clinical course, or PE diagnosis. Site investigators interpreted the 12‐lead ECGs using predefined criteria discussed in pre‐enrollment training modules. The 12‐lead ECG was evaluated for each of the following previously reported patterns in PE: sinus tachycardia (≥100 beats per minute); incomplete right bundle branch block (iRBBB) if QRS complex ≥0.10 and <0.12 seconds and complete right bundle branch block (cRBBB) if QRS complex width ≥0.12 seconds; compilation of S wave in lead I, Q wave in lead III, and T wave inversion (TWI) in lead III, referred to as S1‐Q3‐T3 (the McGinne‐White sign); TWIs in precordial leads V_2–4_; TWI in leads II, III, and aVF; supraventricular tachycardia (SVT; including atrioventricular [AV] nodal reentry tachycardia, AV reentrant tachycardia, atrial tachycardia [unifocal or multifocal], junctional tachycardia, sinus nodal reentry tachycardia, atrial fibrillation, and atrial flutter); ST‐segment depressions (STDs) in V_4–6_; ST‐segment elevation (STE) in lead aVR, left bundle branch block (LBBB); and LV hypertrophy (LVH) with associated TWI.^2^, ^4^, ^5^, ^8^ The ECG was considered positive for abnormal patterns if one or more of the above patterns were present. A TWI wave was defined as a negative deflection of the T wave greater than 0.5 mV below the T–P segment, which represents the isoelectric line (except in aVR or coexisting RBBB). TWI or STE associated with LBBB or RBBB patterns were considered normal. We used predefined criteria and guidelines. STE was determined 1 mm above the J point relative to the TP segment in the absence of early repolarization signs, LVH, or LBBB. ST depression was determined >0.5 mm below the J point relative to the TP segment. TWI or STE in precordial leads associated with LBBB or RBBB patterns were not considered independent ECG abnormalities for this study. In addition, we reported the total number of mutually exclusive abnormal ECG patterns in the patient's initial ECG as a discrete predictor variable.

### Key outcome measures

The primary outcome was the incidence of one or more discrete clinical deterioration events that were considered PE‐related complications, including death. Clinical deterioration included any episode that required hospital‐based monitoring or hospital‐based support or intervention. As previously reported, clinical deterioration endpoints included respiratory failure, cardiac arrest, new dysrhythmia, sustained hypotension requiring intravenous volume expansion or adrenergic medication, and escalated PE interventions.

In this prospective study, each site had institutional guidelines on PE management. We defined respiratory failure as respiratory distress associated with emergent or unplanned mechanical ventilation (intubation, non‐invasive positive pressure ventilation, or cricothyrotomy). We defined cardiac arrest as new episodes of unstable cardiac rhythm or absent electrical activity with advanced cardiac life support for asystole, pulseless electrical activity, ventricular fibrillation, or unstable ventricular tachycardia. We defined new dysrhythmia as the onset of atrial fibrillation with rapid ventricular response, atrial flutter, SVT, stable ventricular tachycardia, or bradycardia that was not present at the ED presentation. We defined sustained hypotension as systolic blood pressure <90 mm Hg (or >40 mm Hg decrease from baseline) or shock index greater than 1.0 for >15 minutes with administration of >499 ml of intravenous fluids bolus for volume expansion or an infusion of norepinephrine, dopamine, or epinephrine. Escalated PE interventions included reperfusion interventions (systemic or catheter‐directed thrombolysis, mechanical catheter directed aspiration, and surgical thrombectomy) and extracorporeal membrane oxygenation interventions.^13^ Clinical deterioration episodes were considered as single discrete episodes if separated by 12 hours or more. Individual patients could have more than one episode of clinical deterioration within the 5‐day window. We followed patients via electronic medical record review during the hospital stay and up to 30 days later, as previously published.^13^

The secondary outcome was the presence of abnlRV as determined by GDE. GDE was performed during the ED visit by emergency medicine physicians trained in GDE image acquisition and subsequently interpreted by site investigators (advanced emergency ultrasound fellowship directors). As previously reported, abnlRV included the presence of one or more of the following echocardiography findings: severe RV dilatation detected as qualitative assessment of RV:LV ratio ≥1:0 and RV apex blunting in two or more views; severe RV systolic dysfunction as qualitatively estimated by diminished longitudinal contraction of the RV (estimated tricuspid annular plane systolic excursion [TAPSE] ≤ 1.0 cm in subcostal or apical four‐chamber views); and flattening or leftward bowing of the interventricular septum.^13^, ^14^ (Rationale for using TAPSE <1.0 cm as cut‐off: The American Society of Echocardiography provides a normal reference range for RV measurements but no stratified classification for mild, moderate, or severe RV dilatation or systolic dysfunction.^15^, ^16^, ^17^ In previous reports, using TAPSE <1.0 cm to estimate severe RV systolic dysfunction had strong agreement with comprehensive echocardiography and strong inter‐ and intra‐rater agreement.)

Site investigators (advanced emergency ultrasound fellowship directors) reviewed and interpreted the images according to interpretation guidelines discussed in training modules before the start of the study. GDE for abnlRV in PE was scored on a 0–3 scale, where 0 = no severe RV dilatation, 1 = severe RV dilatation only, 2 = severe RV dilatation with either septal deviation or severe RV systolic dysfunction, and 3 = severe RV dilatation with both septal deviation and severe RV systolic dysfunction. The presence of abnlRV was defined by a score of 1, 2, or 3. The frequency of ECG patterns was compared to abnlRV responses as an ordinal variable (determined by the GDE severity score). As previously reported in the main manuscript from the PE registry database, the ordinal scale showed increased odds of clinical deterioration as scores increased.^13^ The GDE scale showed high intra‐ and inter‐rater agreement and accuracy compared to comprehensive echocardiography.^14^, ^18^

We compared the mean number of ECG patterns between assigned risk groups within a PE triaging strategy. We stratified each PE triaging strategy into low‐risk and not low‐risk groups. We used three PE triaging strategies: simplified pulmonary embolism severity index (sPESI), modified European Society of Cardiology (ESC), and the pulmonary embolism short‐term clinical outcomes estimator (PE‐SCORE).^13^, ^19^, ^20^ The sPESI was dichotomized as low‐risk (0 points) versus not low‐risk (>0 points). ESC was dichotomized as low‐risk versus not low‐risk. Low‐risk ESC was defined as sPESI = 0 points and being without abnormal cardiac biomarkers or abnormal RV by CT or echocardiography. Not low‐risk PE involves abnormal vital signs, RV abnormality by cardiac biomarkers, or abnormal RV by imaging (CT or echocardiography). PE‐SCORE, which has a scale of 0 to 10 total points, was stratified as low‐risk (0 points), intermediate‐risk (1 to 4 points), and high‐risk (>4 points) groups.

We compared proportions of the specific ECG patterns to the following abnormal heart features: abnormal LV systolic function (<30%), RV abnormality as measured by CT RV:LV ratio ≥1.0, and elevated cardiac biomarkers (definitions follow). As a surrogate of myocardial stretch, brain natriuretic peptide (BNP) elevation was defined by BNP ≥90 pg/ml or N‐terminal BNP ≥500 pg/ml. We used elevated troponin levels as a surrogate of myocardial injury. Toward the last quarter of the enrollment phase, the central site had an institution‐wide change from troponin I to high sensitivity troponin. As previously reported in the first manuscript of the PE registry, at each participating institution, an elevated troponin I was defined as ≥0.07 ng/ml (99% upper reference limit ≥0.028 ng/ml). An elevated high sensitivity troponin was defined as ≥20 pg/ml for males and 12 pg/ml for females (99% upper reference limit range between 24 and 30 pg/ml). We used i‐STAT BNP test cartridge measured in pg/mL and i‐STAT cardiac troponin test cartridge measured in pg/ml (both from Abbott Point of Care, Abbott Park, IL) for troponin I or high sensitivity troponin assay.

### Data analysis

We conducted univariate analyses for the primary and secondary outcomes. For the secondary outcome, GDE score (ordinal from 0–3) was dichotomized to 0 vs. > 0 for ease of interpretation and presentation of results. In addition, we stratified ECG patterns by abnormal LV systolic function, CT RV:LV ratio elevation, troponin elevation, and BNP elevation as binary dependent variables. We stratified the number of abnormal ECG patterns by the three PE triaging strategy classifications as dependent variables. Descriptive statistics included means, standard deviations, median, and interquartile ranges (IQRs) for continuous variables, as well as frequencies and percentages for binary variables. For univariable statistical comparisons, we used two‐sample t‐tests or analysis of variance (ANOVA) for continuous variables and chi‐square tests for categorical variables.

For each primary and secondary outcome, we fit a multivariable logistic regression model with all ECG variables included as primary main effects of interest, and with the following as confounders: race, gender, ethnicity, age, initial heart rate, shock index, initial respiratory rate, initial oxygen saturation, preceding episode of syncope, prior diagnosis of PE, and whether patients had one or more abnormal ECG patterns or not. In addition, in the model for primary composite outcome, we included RV:LV ratio ≥1 as determined by CT, troponin elevation, echocardiography showing abnlRV, and natriuretic peptide elevation as additional predictors. We displayed results for each full regression model in tables with odds ratios (ORs) with 95% confidence intervals (CIs). All *p* values <0.05 were considered statistically significant.

## RESULTS

**FIGURE 1:**
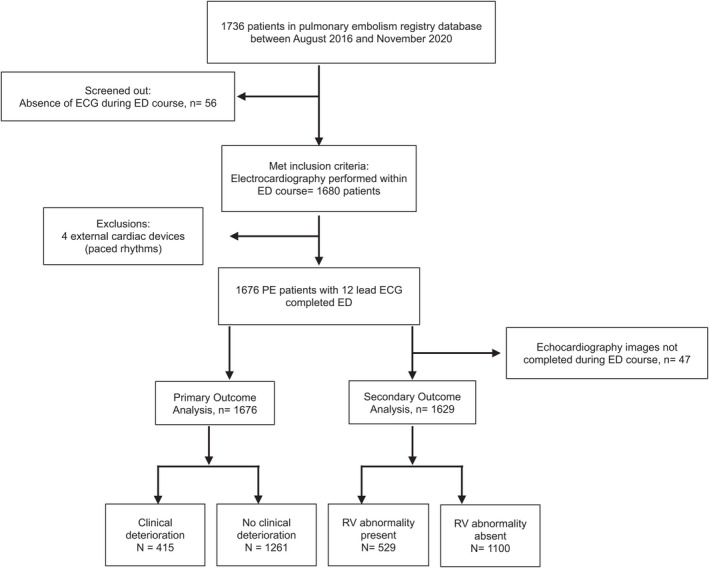
Flow diagram for patients and key outcomes

As shown in Figure 1, 1736 PE patients met inclusion criteria for the registry databases between August 2016 and November 2020. Of these, 1680 (96.8%) patients had an initial ECG performed in the ED. Four of these patients had external cardiac devices leading to ventricularly paced rhythms and were excluded from this analysis. We therefore had 1676 patients with initial native ECG tracings available for statistical analyses for the primary outcome. ECGs were performed within a mean of 1.90 ± 3.38 hours of measuring initial vital signs. Enrollment numbers for the six clinical sites were: Site 1 (695, 41.5%), Site 2 (248, 14.8%), Site 3 (206, 12.3%), Site 4 (156, 9.3%), Site 5 (185, 11.0%), and Site 6 (186, 11.1%).

All ECGs and GDE were performed during the immediate ED course and before anticoagulation or escalated PE intervention. Anticoagulation or escalated PE intervention was initiated either during the ED course or immediately on arrival to the hospital floor in 97.2% (1630/1676) of the PE patients. We followed patients via EMR review during the hospital stay and up to 30 days, as previously published.^13^ All patients included in this report had 5‐day follow up information completed.

Demographics (Table 1) included the mean age of 59.6 ± 16.6 years, 48.2% female, 65.5% Caucasian, 29.1% African American, and 1% Asian. The mean shock index was 0.77 ± 0.25 beats per minute/mm Hg, respiratory rate 19.9 ± 4.6 breaths per minute, and oxygen saturation 95.4 ± 4.6%. Of the 1676 patients, 160 (9.5%) had a preceding episode of syncope and 420 (25.1%) had prior diagnosis of PE or deep venous thrombosis (DVT). For each PE triaging strategy, clinical deterioration was experienced by a lower proportion of patients classified as low‐risk compared to those classified as not low‐risk.

**TABLE 1:** Patient characteristics

	No clinical deterioration (*N* = 1261)	Clinical deterioration (*N* = 415)	Overall (*N* = 1676)
Gender			
Female	601 (47.7%)	206 (49.6%)	807 (48.2%)
Male	660 (52.3%)	209 (50.4%)	869 (51.8%)
Race			
Caucasian	819 (64.9%)	279 (67.2%)	1098 (65.5%)
African/American	376 (29.8%)	112 (27.0%)	488 (29.1%)
American Indian/Alaskan Native	10 (0.8%)	2 (0.5%)	12 (0.7%)
Asian	12 (1.0%)	4 (1.0%)	16 (1.0%)
Ethnicity			
Hispanic	91 (7.2%)	25 (6.0%)	116 (6.9%)
Mean Age, years			
Mean	58.76 ± 16.8	62.2 ± 15.7	59.6 ± 16.6
Systolic blood pressure, mm Hg			
Mean	135.5 ± 23.1	122.2 ± 25.5	132.2 ± 24.4
Heart rate, beats per minute			
Mean	95.4 ± 20.05	106.2 ± 23.6	98.1 ± 21.5
Shock index			
Mean	0.73 ± 0.22	0.91 ± 0.29	0.77 ± 0.25
Respiratory rate, breaths per minute			
Mean	19.6 ± 4.2	21.0 ± 5.54	19.9 ± 4.60
Oxygen saturation, %			
Mean	95.8 ± 4.2	94.2 ± 5.6	95.4 ± 4.63
Cancer			
Present	302 (23.9%)	109 (26.3%)	411 (24.5%)
COPD			
Present	176 (14.0%)	75 (18.1%)	251 (15.0%)
Previous PE/DVT			
Present	313 (24.8%)	107 (25.8%)	420 (25.1%)
Abnormal RV by GDE (*N* = 1629)			
Present	296 (24.2%)	233 (57.5%)	529 (43.2%)
Absent	928 (75.8%)	172 (42.5%)	1100 (89.9%)
PE‐SCORE			
Low‐risk (0 points)	294 (23.3%)	15 (3.6%)	309 (18.4%)
Intermediate‐risk (1–4 points)	864 (68.5%)	295 (71.1%)	1159 (69.2%)
High‐risk (>4 points)	26 (2.1%)	75 (18.1%)	101 (6.0%)
Missing	77 (6.1%)	30 (7.2%)	107 (6.4%)
ESC			
Low‐risk	166 (13.2%)	5 (1.2%)	171 (10.2%)
Not low‐risk	1095 (86.8%)	410(98.8%)	1505 (89.8%)
Missing	0(0%)	0 (0%)	0 (0%)
sPESI			
Low‐risk	518 (41.1%)	71 (17.1%)	589 (35.1%)
Not low‐risk	743 (58.9%)	344 (82.9%)	1087 (64.9%)
Missing	0 (0%)	0 (0%)	0 (0%)

Abbreviations: COPD, chronic obstructive pulmonary disease; DVT, deep venous thrombosis; ESC, European Society of Cardiology PE dichotomized into low‐risk versus not low‐risk classifications; GDE, goal‐directed echocardiography; PE, pulmonary embolism; PE‐SCORE, pulmonary embolism short‐term clinical outcomes risk estimator; RV, right ventricle; sPESI, simplified Pulmonary Embolism Severity Index.

Of the 1676 patients, 415 (24.7%) had one or more clinical deterioration events or hospital‐based intervention within 5 days of PE diagnosis. Forty‐seven of the 1676 patients did not have GDE. Of the 1629 patients with ECG and GDE, 529 (32.4%) had abnlRV by GDE. Table 1 shows numbers and proportions with abnlRV by GDE stratified by clinical deterioration, leading to an OR of 4.25 (3.35, 5.38, *p* < 0.0001).

Table 2 shows the prevalence of each specific ECG pattern. By univariate analysis, the absence of abnormal ECG patterns had an OR for clinical deterioration of 0.34 (0.25, 0.44; *p* < 0.001). The most common ECG patterns were sinus tachycardia (38.9%), S1‐Q3‐T3 pattern (16.3%), incomplete or complete RBBB (15.1%), and TWI in V_2–4_ (14%). In comparison to patients without the specific ECG pattern, those with the following patterns were significantly more likely to experience clinical deterioration: sinus tachycardia, TWI in V_2–4_, STE in aVR, STE in V_1_, SVT, TWI in II and III and aVF, and STD in V_4–6_. LVH with TWI (3%) and LBBB were uncommon and not significantly different between outcome groups.

**TABLE 2:** ECG patterns stratified by primary clinical outcomes^a^

	No clinical deterioration (*N* = 1261)	Clinical deterioration (*N* = 415)	Overall (*N* = 1676)	OR	*p*‐Value
Mean number of abnormal ECG patterns	1.20 ± 1.31	1.99 ± 1.67	1.34 ± 1.44		<0.0001
Number of abnormal ECG patterns					
Zero abnormal patterns	509 (40.4%)	77 (18.6%)	586 (35.0%)	0.34 (0.26, 0.44)	<0.0001
>0 abnormal patterns	751 (59.6%)	338 (81.4%)	1089 (65.0%)		
Missing	1 (0.1%)	0 (0%)	1 (0.1%)		
Complete RBBB					
Absent	1180 (93.6%)	369 (88.9%)	1549 (92.4%)	1.82 (1.24, 2.66)	0.003
Present	81 (6.4%)	46 (11.1%)	127 (7.6%)		
Incomplete RBBB					
Absent	1175 (93.2%)	376 (90.6%)	1551 (92.5%)	1.42 (0.95, 2.11)	0.104
Present	86 (6.8%)	39 (9.4%)	125 (7.5%)		
Sinus tachycardia					
Absent	835 (66.2%)	189 (45.5%)	1024 (61.1%)	2.34 (1.87, 2.94)	<0.001
Present	426 (33.8%)	226 (54.5%)	652 (38.9%)		
S1‐Q3‐T3 pattern					
Absent	1080 (85.6%)	322 (77.6%)	1402 (83.7%)	1.72 (1.30, 2.28)	<0.001
Present	181 (14.4%)	93 (22.4%)	274 (16.3%)		
STE V_1_					
Absent	1162 (92.1%)	362 (87.2%)	1524 (90.9%)	1.72 (1.21, 2.45)	0.003
Present	99 (7.9%)	53 (12.8%)	152 (9.1%)		
T wave inversions V_2–4_ ^b^					
Absent	1112 (88.2%)	330 (79.5%)	1442 (86.0%)	1.92 (1.43, 2.58)	<0.001
Present	149 (11.8%)	85 (20.5%)	234 (14.0%)		
T wave inversions II, III, aVF					
Absent	1153 (91.4%)	354 (85.3%)	1507 (89.9%)	1.84 (1.32, 2.57)	<0.001
Present	108 (8.6%)	61 (14.7%)	169 (10.1%)		
ST‐segment depression V_4–6_					
Absent	1183 (93.8%)	354 (85.3%)	1537 (91.7%)	2.61 (1.83, 3.73)	<0.001
Present	78 (6.2%)	61 (14.7%)	139 (8.3%)		
STE aVR					
Absent	1146 (90.9%)	324 (78.1%)	1470 (87.7%)	2.82 (2.09, 3.82)	<0.001
Present	114 (9.0%)	91 (21.9%)	205 (12.2%)		
Missing	1 (0.1%)	0 (0%)	1 (0.1%)		
SVT (including atrial fibrillation with rapid ventricular response)					
Absent	1218 (96.6%)	361 (87.0%)	1579 (94.2%)	4.24 (2.80, 6.43)	<0.001
Present	43 (3.4%)	54 (13.0%)	97 (5.8%)		
LBBB associated with TWI					
Absent	1238 (98.2%)	412 (99.3%)	1650 (98.4%)	0.39 (0.11, 1.31)	0.179
Present	23 (1.8%)	3 (0.7%)	26 (1.6%)		
LVH with TWI					
Absent	1233 (97.8%)	402 (96.9%)	1635 (97.6%)	1.42 (0.73, 2.78)	0.39
Present	28 (2.2%)	13 (3.1%)	41 (2.4%)		

Abbreviations: ECG, electrocardiography; LBBB, left bundle branch block; LVH, left ventricular hypertrophy; OR, odds ratio; STE, ST‐segment elevation; SVT, supraventricular tachycardia; RBBB, right bundle branch block; TWI, T‐wave inversion (0.5 mV negative deflection).

^a^ The total number of ECG patterns present was greater than the total number of unique patients because any single patient could have more than one of the ECG patterns.

^b^ For those with either iRBBB/cRBBB without TWI in V_2–4_ (*n* = 168) versus those with TWI V_2–4_ (*n* = 82), 56 (33.3%) and 29 (35.2%) had clinical deterioration, respectively. The OR for iRBBB without TWI was 0.91 (0.52, 1.59; *p* = 0.75).

The results of full model multivariable logistic regression analysis are shown in Table 3. SVT was the only ECG pattern that was an independent predictor of CD. Absence of abnormal ECG patterns was not an independent predictor. Abnormal RV by GDE, elevated troponin measurements, elevated natriuretic peptide measurements, CT derived RV:LV ratio ≥1.0, shock index >1.0 and preceding episode of syncope were independent predictors of clinical deterioration. Table 4 shows univariable analysis results for all ECG patterns, except LBBB and LVH, had significant ORs for abnlRV by GDE (our secondary outcome). Sinus tachycardia, TWI in II III, aVF and V_2–4_, S1–Q3‐T3, iRBBB, and STE in aVR had ORs with the lower boundary of 95% CI above 1.0. For presence and absence of abnlRV by GDE, the mean number of abnormal ECG patterns was 2.20 ± 1.69 and 0.93 ± 1.13, respectively (*p* < 0.0001). The absence of abnormal ECG patterns had an OR for abnlRV by GDE of 0.24 (0.18, 0.31; *p* < 0.001). The results of full multivariable logistic regression analysis are shown in Table 5. Independent ECG predictors of abnlRV in order of ORs (high to low) were: STE in aVR, TWI V_2–4_, incomplete RBBB, S1‐Q3‐T3, and sinus tachycardia. LVH with associated TWI lowered the odds of abnlRV. With multivariable analysis, the absence of any of the ECG patterns was not an independent predictor of clinical deterioration. Clinical predictors were age, shock index, initial heart rate, initial respiratory rate, and preceding episode of syncope.

**TABLE 3:** Multivariable analysis stratified by clinical deterioration within 5 days

Predictors	Primary composite outcome (one or more clinical deterioration events within 5 days)		
	OR	CI	*p‐Value*
(Intercept)	0.56	0.03–13.59	0.722
Complete RBBB	1.06	0.64–1.73	0.825
Incomplete RBBB	1.03	0.61–1.69	0.918
Sinus tachycardia	1.43	0.96–2.15	0.081
S1‐Q3‐T3 pattern	0.85	0.58–1.22	0.375
ST elevation V_1_	0.90	0.55–1.45	0.670
T wave inversions V2‐4	1.01	0.66–1.52	0.981
T wave inversions II, III, aVF	1.08	0.67–1.71	0.754
ST depression in V 4–6	1.14	0.69–1.85	0.614
STE aVR	1.16	0.75–1.78	0.494
SVT	2.87	1.66–5.00	<0.001
LBBB with associated TWI	0.21	0.03–0.86	0.060
LVH with associated TWI	1.49	0.60–3.47	0.369
Echocardiography showing abnormal RV	1.68	1.17–2.41	0.005
Elevated troponin level	1.47	1.08–2.00	0.014
RV:LV ratio 1.0 or greater by CT	1.49	1.07–2.08	0.019
Elevated natriuretic peptide level	1.26	0.93–1.70	0.130
Male	1.01	0.77–1.32	0.952
African American/Black	0.66	0.36–1.23	0.182
White	0.81	0.46–1.48	0.486
Ethnicity	1.00	1.00–1.00	0.322
Age	1.01	1.00–1.02	0.025
Initial heart rate	0.99	0.97–1.00	0.008
Initial shock index	13.07	5.69–30.65	<0.001
Initial respiratory rate	1.01	0.98–1.04	0.534
Initial oxygen saturation on room air	0.97	0.95–1.00	0.060
Preceding episode of syncope	1.75	1.14–2.66	0.010
Prior history of PE or DVT	1.02	0.75–1.39	0.885
No abnormal ECG pattern	0.84	0.54–1.32	0.460
Observations	1472		
*R* ^2^ Tjur	0.197		

Abbreviations: CI, confidence interval; DVT, deep venous thrombosis; ECG, electrocardiogram; LBBB, left bundle branch block; LVH, left ventricular hypertrophy; OR, odds ratio; PE, pulmonary embolism; STE, ST‐segment elevation; SVT, supraventricular tachycardia; TWI = T‐wave inversion (0.5 mV negative deflection).

**TABLE 4:** Univariable analysis of ECG patterns stratified by echocardiographic RV abnormalities

	Echocardiography showing RV abnormalities (47 missing)			
	No (*N* = 1100)	Yes (*N* = 529)	OR	*p*‐Value
Mean number of ECG patterns (standard deviation)	0.93 (1.13)	2.20 (1.69)		<0.0001
Number of abnormal ECG patterns				
None	485 (44.1%)	84 (15.9%)	0.24 (0.18, 0.31)	<0.001
One or more	614 (55.8%)	445 (84.1%)		
Missing	1 (0.1%)	0 (0%)		
Complete RBBB				
Absent	1043 (94.8%)	461 (87.1%)	2.70 (1.87, 3.9)	<0.001
Present	57 (5.2%)	68 (12.9%)		
Incomplete RBBB				
Absent	1046 (95.1%)	458 (86.6%)	2.95 (2.04, 4.26)	<0.001
Present	54 (4.9%)	71 (13.4%)		
Sinus tachycardia				
Absent	759 (69.0%)	241 (45.6%)	2.67 (2.15, 3.30)	<0.001
Present	341 (31.0%)	288 (54.4%)		
S1‐Q3‐T3 pattern				
Absent	991 (90.1%)	371 (70.1%)	3.87 (2.95, 5.08)	<0.001
Present	109 (9.9%)	158 (29.9%)		
STE V_1_				
Absent	1026 (93.3%)	454 (85.8%)	2.29 (1.63, 3.22)	<0.001
Present	74 (6.7%)	75 (14.2%)		
T wave inversions V_2–4_				
Absent	1019 (92.6%)	381 (72.0%)	4.89 (3.64, 6.57)	<0.001
Present	81(7.4%)	148 (28.0%)		
T wave inversions II, III, aVF				
Absent	1029 (93.5%)	434 (82.0%)	3.17 (2.29, 4.40)	<0.001
Present	71 (6.5%)	95 (18.0%)		
ST‐segment depression V_4–6_				
Absent	1038 (94.4%)	453 (85.6%)	2.81 (1.98, 4.00)	<0.001
Present	62 (5.6%)	76 (14.4%)		
STE aVR				
Absent	1023 (93.0%)	404 (76.4%)	4.16 (3.06, 5.55)	<0.001
Present	76 (6.9%)	125 (23.6%)		
Missing	1 (0.1%)	0 (0%)		
SVT (including atrial fibrillation with rapid ventricular response)				
Absent	1049 (95.4%)	485 (91.7%)	1.87 (1.23, 2.83)	0.004
Present	51 (4.6%)	44 (8.3%)		
LBBB associated with TWI				
Absent	1079 (98.1%)	524 (99.1%)	0.49 (0.18, 1.31)	0.214
Present	21 (1.9%)	5 (0.9%)		
LVH with TWI				
Absent	1070 (97.3%)	518 (97.9%)	0.76 (0.38, 1.52)	0.54
Present	30 (2.7%)	11 (2.1%)		

Abbreviations: ECG, electrocardiogram; LBBB, left bundle branch block; LVH, left ventricular hypertrophy; OR, odds ratio; RBBB, right bundle branch block; RV, right ventricle; STE, ST‐segment elevation; SVT, supraventricular tachycardia; TWI, T‐wave inversion (0.5 mV negative deflection).

**TABLE 5:** Full multivariable analysis model stratified by RV abnormalities (by echocardiography)

Predictors	Echocardiography with abnlRV		
	OR	CI	*p*‐Value
(Intercept)	30.78	1.09–931.83	0.046
Complete RBBB	1.23	0.74–2.04	0.422
Incomplete RBBB	2.63	1.59–4.38	<0.001
Sinus tachycardia	1.68	1.14–2.49	0.009
S1‐Q3‐T3 pattern	2.42	1.70–3.47	<0.001
STE V_1_	1.36	0.83–2.22	0.215
T wave inversions V_2–4_	4.00	2.64–6.12	<0.001
T wave inversions II, III, aVF	1.26	0.78–2.02	0.349
ST depression in V_4–6_	0.79	0.47–1.31	0.364
STE aVR	2.42	1.58–3.74	<0.001
SVT	1.25	0.71–2.18	0.441
LBBB with associated TWI	0.30	0.07–0.97	0.069
LVH with associated TWI	0.33	0.11–0.86	0.029
Male	0.97	0.75–1.27	0.848
African American/Black	1.18	0.64–2.25	0.605
White	1.05	0.58–1.95	0.883
Ethnicity	1.00	1.00–1.00	0.579
Age	1.02	1.01–1.03	<0.001
Initial heart rate	1.00	0.99–1.01	0.664
Initial shock index	5.45	2.36–12.76	<0.001
Initial respiratory rate	1.04	1.02–1.08	0.003
Initial oxygen saturation on room air	0.92	0.89–0.94	<0.001
Preceding episode of syncope	3.09	2.00–4.82	<0.001
Prior history of PE or DVT	1.07	0.79–1.44	0.651
No abnormal ECG pattern	1.08	0.70–1.68	0.722
Observations	1472		
*R* ^2^ Tjur	0.284		

Abbreviations: CI, confidence interval; DVT, deep venous thrombosis; ECG, electrocardiogram; LBBB, left bundle branch block; LVH, left ventricular hypertrophy; OR, odds ratio; PE, pulmonary embolism; RBBB, right bundle branch block; STE, ST‐segment elevation; SVT, supraventricular tachycardia; TWI, T‐wave inversion (0.5 mV negative deflection).

Of the 1676 patients, 1090 (65.0%) had at least one of the abnormal ECG patterns. The mean number of abnormal ECG patterns was 1.33 ± 1.4. For patients with and without the primary outcome, the mean numbers of abnormal ECG patterns were 1.97 ± 1.65 and 1.20 ± 1.30, respectively (Table 2). Supporting Information Table S1, which is available online, shows patients with low‐risk classifications by all three PE triaging strategies had a lower number of abnormal ECG patterns compared to those in the higher risk classifications.

Supporting Information Tables S2–S9 show results of analyses of specific ECG patterns stratified by LV systolic dysfunction, RV dilatation by CT, and elevated cardiac biomarkers. Of 1629 with GDE, 1620 had a report on LV systolic function, with 127 (7.8%) having severe LV dysfunction. SVT, LBBB, and LVH with TWI were significantly associated with severe LV systolic dysfunction by univariable analysis (Supporting Information Table S2), whereas only SVT and LBBB were significant by multivariable analysis (Supporting Information Table S3). Supporting Information Table S4 shows 450 of 1659 patients (27.1%) had troponin elevations. All ECG patterns, except SVT, LBBB, and LVH with TWI, were associated with troponin elevation by univariable analysis. However, Supporting Information Table S5 shows sinus tachycardia, S1‐Q3‐T3, TWI V_2–4_, and STE aVR were independent predictors of troponin elevations by multivariable analysis. Supporting Information Table S6 shows 642 of 1608 patients (39.9%) with BNP measurements had BNP elevations. All ECG patterns, except iRBBB, were associated with natriuretic peptide elevation by univariable analysis. Supporting Information Table S7 shows S1‐Q3‐T3, STE V_1_, TWI V_2–4_, and SVT were significant by multivariable analysis. Supporting Information Table S8 shows elevated CT RV:LV ratios in 552 of 1640 patients (33.7%) who had CT performed. All ECG patterns, except SVT, LBBB, and LVH with TWI, were associated with elevated CT RV:LV ratio by univariable analysis. Supporting Information Table S9 shows complete and incomplete RBBB, S1‐Q3‐T3, TWI V_2–4_, STE aVR, and SVT were significant by multivariable analysis. Finally, Supporting Information Table S10 shows the prognostic metrics of the following categorical variables: abnormal ECG patterns, elevated troponin, elevated natriuretic peptide, initial shock index (>1.0), CT RV:LV ratio, hypotension (< 100 mm Hg), hypoxia (<92%), and preceding syncope. Predictor positive likelihood ratios ordered high to low were: hypotension, initial shock index, preceding syncope, hypoxia, elevated troponin, increased RV:LV ratio, elevated natriuretic peptide, and abnormal ECG pattern. The number of abnormal ECG patterns and the CT RV:LV ratio demonstrated the highest negative predictive values, followed by elevated natriuretic peptide, initial shock index, elevated troponin, hypotension, hypoxia, and preceding syncope.

### LIMITATIONS

This study had several limitations. First, preexisting ECG findings were not reviewed to determine if index PE‐associated ECG abnormalities were acute or preexisting. Second, there are multiple causes of tachycardia that are unrelated to and not directly provoked by the pathophysiology of PE. Third, TWIs may be associated with normal variants and can be associated with RBBB. Furthermore, the RBBB pattern can exist in the asymptomatic population without PE.^21^ Fourth, we reported predetermined ECG patterns that were fully recorded and part of the medical record. It is plausible that ECG abnormalities were in evolution. STE was defined as the ST segment being ≥1 mm above the isoelectric line (TP segment). However, use of the J point relative to PQ junction, instead of TP segment, is currently advised for determining ST depression.^22^

Several investigators witnessed important but transient changes to ECG patterns in higher acuity acute PE patients that were not fully recorded or available in the EMR for interpretation or analysis. Our report focused on the initial ECG in the ED; however, there were several instances in patients with abnormal RV by GDE where conduction, TWI, or ST‐segment abnormalities were transient. We observed several instances of normalization of initial abnormal ECG patterns and improvements to RV dilatation or systolic function (determined by serial echocardiography) after administration of thrombolytic agents. Further supporting evidence for this observation is provided by Choi et al., who showed not only an association between TWI and abnlRV, but also found that reversals of T wave abnormalities were temporally associated with resolution of an abnlRV.^23^ Other ECG patterns pertinent to PE, such as isolated TWI in III and V_1_, were not included in this report because of lack of prognostic significance. In our database, the TWI III and V_1_ pattern was present in 17.6% (73/415) and 17.4% (219/1261) of those with and without clinical deterioration, respectively. Finally, we did not examine the prevalence of specific ECG patterns in patients without acute PE to compare with prevalence in patients with PE.

## DISCUSSION

We prospectively evaluated PE patients in the ED to report on the associations of multiple specific ECG patterns for clinical deterioration and abnlRV by echocardiography using univariable and multivariable analyses. ECGs were performed early and contemporaneously with GDE assessments for abnlRV during the ED work‐up. Abnormal RV (as determined by GDE) had an OR of 4.25 (3.35, 5.38) for clinical deterioration. The absence of abnormal ECG patterns on initial early ECG had ORs of 0.34 (0.26, 0.44) and 0.24 (0.18, 0.31) for clinical deterioration and abnlRV by GDE, respectively. However, on multivariable analysis, absence of any ECG patterns did not qualify as an independent predictor of clinical deterioration or abnlRV.

We found all abnormal ECG patterns had statistically significant increased ORS above unity for clinical deterioration within 5 days of PE diagnosis, except for LBBB and LVH with TWI. Of the ECG patterns, only SVT was significant as an independent predictor for clinical deterioration. For abnlRV by echocardiography, associations were significant for most ECG patterns. Sinus tachycardia, STE in aVR, incomplete RBBB, S1‐Q3‐T3, and TWI V_2–4_ were significant as independent predictors for abnlRV by GDE. Patients with acute pulmonary embolism may have one or more of the specific ECG abnormalities. In patients with confirmed acute pulmonary embolism, one or more of these ECG patterns should increase considerations for performance of echocardiography to look for RV abnormalities and, if present, increase concerns for early clinical deterioration. By univariable analysis, we found the absence of any of the abnormal ECG patterns was associated with lower probability of clinical deterioration and abnlRV by GDE. However, with full multivariable analysis with other ECG and non‐ECG variables, the number of abnormal ECG patterns was not an independent predictor of clinical deterioration or abnlRV by echocardiography.

Most previous studies have been retrospective analyses with ECGs completed 10 to 72 hours removed from the time of PE diagnosis. Strengths of our study include its prospective design, a large database of patients representing the full spectrum of PE severity, and early timing of ECG relative to ED diagnosis of PE.

### Clinical deterioration

Previous reports have shown several ECG patterns (STE, STsegment depression, S1‐Q3‐T3, RBBB, TWI, and SVT) were associated with clinical deterioration.^2^, ^12^ Similarly, by univariable analysis, our prospective study showed significant associations of these abnormal ECG patterns with clinical deterioration within 5 days. In addition, we noted the following ECG patterns were also associated with increased risk for clinical deterioration: STE in V_1_; TWI in leads II, III, and aVF; ST depression in V_4_–V_6_; STE in aVR; and SVT (Table 2). However, SVT was the only ECG pattern determined to be an independent predictor from multivariable analyses in the presence of other clinical variables.

### Abnormal RV by echocardiography

In our study, early echocardiography findings of abnormal RV within the ED course were significantly associated with clinical deterioration within 5 days. In turn, abnormal ECG patterns (sinus tachycardia, STE in aVR, incomplete RBBB, S1‐Q3‐T3, and TWI V_2–4_) were independent predictors of abnlRV by echocardiography even in the presence of other clinical variables. By multivariable analyses, the number of abnormal ECG patterns was not an independent predictor of clinical deterioration or abnl RV by echocardiography.

Reports on the associations between ECG and abnlRV by echocardiography in PE patients are mixed, with important differences in timing of ECG performance studies, patient cohorts, and the ECG patterns selected. A report by Vanni et al. studied ECG and echocardiography performed within 1 hour of PE diagnosis and found that patients with RBBB, precordial repolarization abnormalities, or the S1‐Q3‐T3 pattern complex were twice as likely to have echocardiographic signs of abnlRV than patients without any of the evaluated ECG patterns.^11^ Punukollu et al. showed S1‐Q3‐T3 and TWI in V_1–3_ were more likely in abnlRV, especially if echocardiography was performed at admission or within 24 hours of hospitalization.^9^ Daniel et al. showed that having multiple abnormal ECG patterns offered low sensitivity but high specificity for echocardiographic evidence of severe abnlRV.^4^ In contrast, another report showed having a few abnormal ECG patterns had moderate sensitivity and moderate specificity for abnormal RV.^10^ In one study, TWI V_2–4_ was six times more frequent in PE patients with abnlRV than those without abnlRV.^6^ Another report showed TWI in V_1–3_, tachycardia, and S wave in lead I were strong predictors of abnlRV by echocardiography, CT, or cardiac biomarkers.^24^ In contrast, a retrospective study of 289 patients by Stein et al. did not find ECG to be useful in predicting the presence or absence of RV dilatation.^3^ In our study, the independent predictors of abnlRV by echocardiography were sinus tachycardia, S1‐Q3‐T3, TWI V_2–4_, and STE in aVR. Our study findings suggest the presence of one or more of these ECG patterns may be associated with the presence of concurrent abnlRV in suspected or confirmed acute PE patients, which in turn, warns providers the PE patient under their care has an increased risk of clinical deterioration within 5 days. The presence of an acute abnlRV may be determined by reviewing recently performed imaging (CT or echocardiography).

Reports on the prevalence of specific PE‐associated ECG abnormalities vary. In a meta‐analysis of 45 studies that involved 8209 PE patients, the most prevalent abnormal ECG patterns were sinus tachycardia (38%), TWI V_1–4_ (38%), and STE in aVR (36%).^12^ Similarly, sinus tachycardia was the most prevalent ECG pattern in our study. Other similarities and differences between our results and previous studies are as follows. First, the prevalence of RBBB (complete or incomplete) in other studies has ranged from 4.8% to 32.0%.^2^ In our study, complete RBBB and incomplete RBBB were present in 7.6% and 7.5% of 1676 patients, respectively. Second, the prevalence of precordial TWI in other studies has ranged from 14% to 59%.^3^, ^4^, ^6^, ^11^, ^16^ In our study, TWI in V_2_–V_4_ was present in 11% of our patients. Third, the prevalence of TWI in inferior leads was as high as 48% of patients in other studies,^16^ whereas only 7% of our patients had inferior TWI. Fourth, previous reports had high prevalence of STE in aVR (34%–45%) in patients with more severe PE presentation, including hypotension, need for thrombolysis or adrenergic medication use.^5^, ^7^ Although we found STE in aVR in less PE patients (12%) than previous studies, STE aVR had ORs of 2.82 for 5‐day clinical deterioration and 4.16 for abnlRV in our study. We also found a prevalence of 18% for the S1‐Q3‐T3 pattern, whereas previous studies have reported a prevalence between 8.5% and 33.0%, and as high as 53% in PE patients with clinical shock. Like previous ECG scoring systems, sinus tachycardia and anterior precordial TWI (V_2–4_) were strong predictors of clinical deterioration.

By univariate analysis, mean number of abnormal ECG patterns was significantly greater in those with clinical deterioration and abnl RV by echocardiography versus those without. The mean number of abnormal ECG patterns was lower in those with low‐risk classification by any of the PE triaging strategies.

## CONCLUSIONS

Several ECG patterns are significantly more frequent in those with clinical deterioration versus those without. SVT was the independent predictor of subsequent clinical deterioration. Sinus tachycardia, STE in aVR, incomplete RBBB, S1‐Q3‐T3, and TWI V_2–4_ were independent predictors of abnlRV by echocardiography. The absence of any of the abnormal ECG patterns was associated with reduced risk for both clinical deterioration and abnlRV by univariable analysis but not by multivariable analysis. By univariate analyses, higher mean number of abnormal ECG patterns was associated with presence of clinical deterioration, RV abnormality by echocardiography, and PE triaging strategy classification as “not low‐risk” versus the groups without these characteristics.

## FUNDING INFORMATION

This was supported by the Agency for Healthcare Research and Quality [grant number R01HS025979]. The content is solely the responsibility of the authors and does not necessarily represent the official views of the Agency for Healthcare Research and Quality.

## AUTHOR CONTRIBUTIONS

A.J.W. designed the study. J.D.R., A.T., C.C., K.L., and A.J.W. supervised the conduct of the trial and data collection. All PESCOR investigators enrolled patients. N.O. and A.J.W. provided statistical analysis and interpretation of the data. A.J.W. managed the data, including quality control. A.J.W., J.D.R., K.L. drafted the manuscript. All authors contributed substantially to article revision for important intellectual content. A.J.W. takes responsibility for the paper as a whole.

## CONFLICT OF INTEREST

All authors report no conflicts of interest.

## Supporting information


Data S1
