# Supplementary material for: Electrocardiographic findings associated with early clinical deterioration in acute pulmonary embolism
Source: Acad Emerg Med. 2022 Jul 20;29(10):1185–96. doi: 10.1111/acem.14554 (PMC9796434; doi:10.1111/acem.14554)
Supplement: Supplementary file 1 — Data S1 [file ACEM-29-1185-s001.zip › ACEM_14554_Table _S6_Final.pdf]

Table S6: Univariable analysis of ECG findings by natriuretic peptide elevation

	Brain natriuretic peptide elevation		
	**No (N = 966)**	**Yes (N = 642)**	**P-value**
**Complete RBBB**			
Absent	908 (94.0%)	575 (89.6%)	0.002
Present	58.0 (6.0%)	67.0 (10.4%)	
**Incomplete RBBB**			
Absent	903 (93.5%)	584 (91.0%)	0.076
Present	63.0 (6.5%)	58.0 (9.0%)	
**Sinus tachycardia**			
Absent	617 (63.9%)	359 (55.9%)	0.002
Present	349 (36.1%)	283 (44.1%)	
**S1-Q3-T3 pattern**			
Absent	837 (86.6%)	506 (78.8%)	<0.001
Present	129 (13.4%)	136 (21.2%)	
**ST elevation V_1**			
Absent	908 (94.0%)	553 (86.1%)	<0.001
Present	58 (6.0%)	89 (13.9%)	
**T-wave inversions V_{2-4}**			
Absent	878 (90.9%)	505 (78.7%)	<0.001
Present	88 (9.1%)	137 (21.3%)	
**T-wave inversions II, III, aVF**			
Absent	897 (92.9%)	548 (85.4%)	<0.001
Present	69 (7.1%)	94 (14.6%)	
**ST segment depression V_{4-6}**			
Absent	897 (92.9%)	577 (89.9%)	0.043
Present	69 (7.1%)	65 (10.1%)	

**ST segment elevation aVR**			
Absent	871 (90.2%)	538 (83.8%)	<0.001
Present	95 (9.8%)	103 (16.0%)	
Missing	0 (0%)	1 (0.2%)	
**SVT** (including atrial fibrillation with rapid ventricular response)			
Absent	941 (97.4%)	574 (89.4%)	<0.001
Present	25 (2.6%)	68 (10.6%)	
**LBBB associated with TWI**			
Absent	957 (99.1%)	626 (97.5%)	0.023
Present	9 (0.9%)	16 (2.5%)	
**LVH with TWI**			
Absent	950 (98.3%)	619 (96.4%)	0.022
Present	16 (1.7%)	23 (3.6%)	

* Abbreviations: LBBB = left bundle branch block; LVH = Left ventricular hypertrophy; RBBB = right bundle branch block; SVT = supraventricular tachycardia (including atrial fibrillation with rapid ventricular response [100 per minute]); TWI = T-wave inversion (0.5 mV negative deflection)
