# Supplementary material for: Electrocardiographic findings associated with early clinical deterioration in acute pulmonary embolism
Source: Acad Emerg Med. 2022 Jul 20;29(10):1185–96. doi: 10.1111/acem.14554 (PMC9796434; doi:10.1111/acem.14554)
Supplement: Supplementary file 1 — Data S1 [file ACEM-29-1185-s001.zip › ACEM_14554_Table S1.pdf]

Table S1. Mean number of abnormal ECG patterns stratified by risk classification for each PE triaging strategy

PE triaging strategy*	Risk classification				
**sPESI**	**Overall** (N = 1676)	**Low-risk** (N = 1011)	**Not low-risk** (N = 558)		**P-value**
Mean number of abnormal ECG patterns ± standard deviation	1.34 ± 1.45	0.89 ± 1.23	1.57 ± 1.50		<0.001
**ESC**	**Overall** (N = 1676)	**Low-risk** (N = 1402)	**Not low-risk** (N = 167)		**P-value**
Mean number of abnormal ECG patterns ± standard deviation	1.34 ± 1.45	0.56 ± 1.0	1.42 ± 1.46		<0.001
**PE-SCORE**	**Overall** (N = 1676)	**Low-risk** 0 points (N = 309)	**Intermediate-risk** 1–4 points (N = 1159)	**High-risk** > 4 points (N = 101)	**P-value**
Mean number of abnormal ECG patterns ± standard deviation	1.34 ± 1.45	0.57 ± 0.95	1.42 ± 1.41	2.61 ± 1.78	<0.001
Missing	1 (0.1%)	0 (0%)	1 (0.1%)	0 (0%)	

* sPESI = simplified pulmonary embolism severity index, ESC = European Society of Cardiology (dichotomized into low-risk versus not low-risk classifications), PE-SCORE = pulmonary embolism short-term clinical outcomes risk estimator
