# Supplementary material for: Electrocardiographic findings associated with early clinical deterioration in acute pulmonary embolism
Source: Acad Emerg Med. 2022 Jul 20;29(10):1185–96. doi: 10.1111/acem.14554 (PMC9796434; doi:10.1111/acem.14554)
Supplement: Supplementary file 1 — Data S1 [file ACEM-29-1185-s001.zip › ACEM_14554_Table S10_Final.pdf]

**Table S10:** Prognostic metrics of categorical variables*

| Predictors | Sensitivity (CI) | Specificity (CI) | Positive Likelihood Ratio | Negative Likelihood Ratio | Positive Predictive Value | Negative Predictive Value |
|---|---|---|---|---|---|---|
| **Abnormal ECG patterns (zero vs. 1 or more)** | 81.45% (77.37–85.07) | 40.40% (37.67–43.17) | 1.37 (1.28–1.46) | 0.46 (0.37–0.57) | 31.04% (29.67–32.44) | 86.86% (84.24–89.10) |
| **Elevated troponin** | 45.15% (40.27–50.09) | 78.83% (76.46–81.07) | 2.13 (1.83–2.48) | 0.70 (0.63–0.76) | 41.33% (37.73–45.04) | 81.31% (79.86–82.67) |
| **Elevated natriuretic peptide** | 56.72% (51.71–61.62) | 65.67% (62.91–68.35) | 1.65 (1.47–1.85) | 0.66 (0.59–0.74) | 35.51% (32.91–38.21) | 81.99% (80.16–83.68) |
| **Initial shock index (> 1.0)** | 39.76% (35.02–44.65) | 86.52% (84.51–88.36) | 2.95 (2.46–3.54) | 0.70 (0.64–0.76) | 49.25% (44.69–53.83) | 81.36% (80.10–82.56) |
| **CT RV:LV Ratio** | 53.96% (48.96–58.90) | 72.98% (70.41–75.44) | 2.00 (1.76–2.27) | 0.63 (0.56–0.70) | 39.49% (36.47–42.60) | 82.90% (81.28–84.42) |
| **Hypotension (< 100 mmHg)** | 19.52% (15.81–23.66) | 95.40% (94.09–96.49) | 4.24 (3.09–5.83) | 0.84 (0.80–0.89) | 58.27% (50.39–65.75) | 78.27% (77.43–79.09) |
| **Hypoxia (< 92%)** | 23.61% (19.61–28.00) | 90.01% (88.22–91.61) | 2.36 (1.86–3.00) | 0.85 (0.80–0.90) | 43.75% (37.97–49.71) | 78.17% (77.19–79.12) |
| **Preceding syncope** | 18.07% (14.49–22.12) | 93.26% (91.73–94.58) | 2.68 (2.01–3.58) | 0.88 (0.84–0.92) | 46.88% (39.77–54.11) | 77.57% (76.73–78.39) |

* Abbreviations: CI = confidence interval, CT = computed tomography, RV = right ventricle, LV = left ventricle
