# Supplementary material for: Electrocardiographic findings associated with early clinical deterioration in acute pulmonary embolism
Source: Acad Emerg Med. 2022 Jul 20;29(10):1185–96. doi: 10.1111/acem.14554 (PMC9796434; doi:10.1111/acem.14554)
Supplement: Supplementary file 1 — Data S1 [file ACEM-29-1185-s001.zip › ACEM_14554_Table S2.pdf]

Table S2: Univariable analysis of ECG findings by left ventricular systolic function

	Severe LV systolic function <30% ejection fraction by echocardiography		
	**No (N = 1493)**	**Yes (N = 127)**	**P-value**
**Complete RBBB**			
Absent	1385 (92.8%)	112 (88.2%)	0.09
Present	108 (7.2%)	15 (11.8%)	
**Incomplete RBBB**			
Absent	1376 (92.2%)	120 (94.5%)	0.44
Present	117 (7.8%)	7 (5.5%)	
**Sinus tachycardia**			
Absent	919 (61.6%)	77 (60.6%)	0.912
Present	574 (38.4%)	50 (38.8%)	
**S1-Q3-T3 pattern**			
Absent	1249 (83.7%)	107 (84.3%)	0.961
Present	244 (16.3%)	20 (15.7%)	
**ST elevation V_1**			
Absent	1360 (91.1%)	113 (89.0%)	0.525
Present	133 (8.9%)	14 (11.0%)	
**T-wave inversions V_{2-4}**			
Absent	1288 (86.3%)	105 (82.7%)	0.324
Present	205 (13.7%)	22 (17.3%)	
**T-wave inversions II, III, aVF**			
Absent	1344 (90.0%)	112 (88.2%)	0.615
Present	149 (10.0%)	15 (11.8%)	
**ST segment depression V_{4-6}**			

Absent	1368 (91.6%)	113 (89.0%)	0.39
Present	125 (8.4%)	14 (11.0%)	
**ST segment elevation aVR**			
Absent	1306 (87.5%)	114 (89.8%)	0.552
Present	186 (12.5%)	13 (10.2%)	
Missing	1 (0.1%)	0 (0%)	
**SVT** (including atrial fibrillation with rapid ventricular response)			
Absent	1420 (95.1%)	105 (82.7%)	<0.001
Present	73 (4.9%)	22 (17.3%)	
**LBBB associated with TWI**			
Absent	1479 (99.1%)	115 (90.6%)	<0.001
Present	14 (0.9%)	12 (9.4%)	
**LVH with TWI**			
Absent	1465 (98.1%)	115 (90.6%)	<0.001
Present	28 (1.9%)	12 (9.4%)	

* Abbreviations: LBBB = left bundle branch block; LVH = Left ventricular hypertrophy; RBBB = right bundle branch block; SVT = supraventricular tachycardia (including atrial fibrillation with rapid ventricular response [100 per minute]); TWI = T-wave inversion (0.5 mV negative deflection)
