# Supplementary material for: Electrocardiographic findings associated with early clinical deterioration in acute pulmonary embolism
Source: Acad Emerg Med. 2022 Jul 20;29(10):1185–96. doi: 10.1111/acem.14554 (PMC9796434; doi:10.1111/acem.14554)
Supplement: Supplementary file 1 — Data S1 [file ACEM-29-1185-s001.zip › ACEM_14554_Table S8.pdf]

Table S8: Univariable analysis of ECG findings by computed tomography right ventricle dilatation

	CT RV:LV ratio elevated		
	**No (N = 1088)**	**Yes (N = 552)**	**P-value**
**Complete RBBB**			
Absent	1037 (95.3%)	482 (87.3%)	<0.001
Present	51 (4.7%)	70 (12.7%)	
**Incomplete RBBB**			
Absent	1030 (94.7%)	489 (88.6%)	<0.001
Present	58 (5.3%)	63 (11.4%)	
**Sinus tachycardia**			
Absent	719 (66.1%)	283 (51.3%)	<0.001
Present	369 (33.9%)	269 (48.7%)	
**S1-Q3-T3 pattern**			
Absent	982 (90.3%)	391 (70.8%)	<0.001
Present	106 (9.7%)	161 (29.2%)	
**ST elevation V_1**			
Absent	1016 (93.4%)	479 (86.8%)	<0.001
Present	72 (6.6%)	73 (13.2%)	
**T-wave inversions V_{2-4}**			
Absent	989 (90.9%)	426 (77.2%)	<0.001
Present	99.0 (9.1%)	126 (22.8%)	
**T-wave inversions II, III, aVF**			
Absent	1006 (92.5%)	470 (85.1%)	<0.001
Present	82 (7.5%)	82 (14.9%)	
**ST segment depression V_{4-6}**			
Absent	1018 (93.6%)	487 (88.2%)	<0.001

Present	70 (6.4%)	65 (11.8%)	
**ST segment elevation aVR**			
Absent	1013 (93.1%)	425 (77.0%)	<0.001
Present	74 (6.8%)	127 (23.0%)	
Missing	1 (0.1%)	0 (0%)	
**SVT** (including atrial fibrillation with rapid ventricular response)			
Absent	1025 (94.2%)	519 (94.0%)	0.967
Present	63 (5.8%)	33 (6.0%)	
**LBBB associated with TWI**			
Absent	1070 (98.3%)	545 (98.7%)	0.696
Present	18 (1.7%)	7 (1.3%)	
**LVH with TWI**			
Absent	1064 (97.8%)	540 (97.8%)	1
Present	24 (2.2%)	12 (2.2%)	

* Abbreviations: CT = computed tomography, RV = right ventricle, LV = left ventricle, RBBB = right bundle branch block, LBBB = left bundle branch block; LVH = left ventricular hypertrophy, SVT = supraventricular tachycardia (including atrial fibrillation with rapid ventricular response [100 per minute]), TWI = T-wave inversion (0.5 mV negative deflection)
