# Supplementary material for: Electrocardiographic findings associated with early clinical deterioration in acute pulmonary embolism
Source: Acad Emerg Med. 2022 Jul 20;29(10):1185–96. doi: 10.1111/acem.14554 (PMC9796434; doi:10.1111/acem.14554)
Supplement: Supplementary file 1 — Data S1 [file ACEM-29-1185-s001.zip › ACEM_14554_Table_S3_Final.pdf]

Table S3: Multivariable analysis of ECG findings by left ventricle systolic dysfunction*

	Severe LV systolic function		
Predictors	*Odds Ratios*	*Confidence Interval*	*p*
(Intercept)	0.92	0.02–45.76	0.967
Complete RBBB	1.56	0.76–3.06	0.204
Incomplete RBBB	0.75	0.27–1.76	0.546
Sinus tachycardia	0.80	0.45–1.42	0.454
**S1-Q3-T3 pattern**	0.87	0.47–1.56	0.659
**ST elevation V_1**	0.91	0.41–1.92	0.818
T wave inversions V_{2-4}	1.18	0.62–2.16	0.602
T wave inversions II, III, aVF	1.17	0.55–2.35	0.671
ST depression in V_{4-6}	1.41	0.64–2.93	0.373
ST segment elevation aVR	0.52	0.23–1.11	0.109
SVT	3.94	2.00–7.59	**<0.001**
Left bundle branch block with associated TWI	14.06	5.28–38.03	**<0.001**

LVH with associated TWI	4.95	1.99–11.73	**<0.001**
Male	0.51	0.33–0.77	**0.002**
African American/Black	1.23	0.55–3.10	0.635
White	0.54	0.24–1.36	0.160
Ethnicity	1.00	1.00–1.00	0.609
Age	1.01	0.99–1.02	0.472
Initial heart rate	1.00	0.98–1.01	0.559
Initial shock index	1.13	0.34–3.62	0.836
Initial respiratory rate	1.07	1.03–1.11	**<0.001**
Initial oxygen saturation on room air	0.96	0.93–1.00	**0.042**
Preceding episode of syncope	0.86	0.39–1.74	0.696
Prior history of PE or DVT	1.26	0.80–1.96	0.313
No abnormal ECG pattern	0.85	0.44–1.63	0.619
Observations	1472		
R2 Tjur	0.108		

* Abbreviations: LV = left ventricle, LBBB = left bundle branch block, LVH = left ventricular hypertrophy, RBBB = right bundle branch block, SVT = supraventricular tachycardia (including atrial fibrillation with rapid ventricular response [100 per minute]), TWI = T-wave inversion (0.5 mV negative deflection)
