# Supplementary material for: Electrocardiographic findings associated with early clinical deterioration in acute pulmonary embolism
Source: Acad Emerg Med. 2022 Jul 20;29(10):1185–96. doi: 10.1111/acem.14554 (PMC9796434; doi:10.1111/acem.14554)
Supplement: Supplementary file 1 — Data S1 [file ACEM-29-1185-s001.zip › ACEM_14554_Table_S4_Final.pdf]

Table S4: Univariable analysis of ECG findings by troponin elevation

Troponin elevation (Complete analysis N = 1659)			
	**No (N = 1209)**	**Yes (N = 450)**	**P-value**
**Complete RBBB**			
Absent	1138 (94.1%)	397 (88.2%)	<0.001
Present	71 (5.9%)	53 (11.8%)	
**Incomplete RBBB**			
Absent	1132 (93.6%)	404 (89.8%)	0.011
Present	77 (6.4%)	46 (10.2%)	
**Sinus tachycardia**			
Absent	822 (68.0%)	196 (43.6%)	<0.001
Present	387 (32.0%)	254 (56.4%)	
**S1-Q3-T3 pattern**			
Absent	1061 (87.8%)	328 (72.9%)	<0.001
Present	148 (12.2%)	122 (27.1%)	
**ST elevation V_1**			
Absent	1121 (92.7%)	387 (86.0%)	<0.001
Present	88 (7.3%)	63 (14.0%)	
**T-wave inversions V_{2-4}**			
Absent	1091 (90.2%)	338 (75.1%)	<0.001
Present	118 (9.8%)	112 (24.9%)	
**T-wave inversions II, III, aVF**			
Absent	1123 (92.9%)	368 (81.8%)	<0.001
Present	86 (7.1%)	82 (18.2%)	
**ST segment depression V_{4-6}**			
Absent	1137 (94.0%)	385 (85.6%)	<0.001

Present	72 (6.0%)	65 (14.4%)	
**ST segment elevation aVR**			
Absent	1106 (91.5%)	351 (78.0%)	<0.001
Present	102 (8.4%)	99 (22.0%)	
Missing	1 (0.1%)	0 (0%)	
**SVT** (including atrial fibrillation with rapid ventricular response)			
Absent	1147 (94.9%)	416 (92.4%)	0.078
Present	62 (5.1%)	34 (7.6%)	
**LBBB associated with TWI**			
Absent	1192 (98.6%)	443 (98.4%)	1
Present	17 (1.4%)	7 (1.6%)	
**LVH with TWI**			
Absent	1185 (98.0%)	435 (96.7%)	0.153
Present	24 (2.0%)	15 (3.3%)	

* Abbreviations: LBBB = left bundle branch block, LVH = left ventricular hypertrophy, RBBB = right bundle branch block, SVT = supraventricular tachycardia (including atrial fibrillation with rapid ventricular response [100 per minute]), TWI = T-wave inversion (0.5 mV negative deflection)
