# Supplementary material for: Electrocardiographic findings associated with early clinical deterioration in acute pulmonary embolism
Source: Acad Emerg Med. 2022 Jul 20;29(10):1185–96. doi: 10.1111/acem.14554 (PMC9796434; doi:10.1111/acem.14554)
Supplement: Supplementary file 1 — Data S1 [file ACEM-29-1185-s001.zip › ACEM_14554_Table_S5_Final.pdf]

Table S5: Full multivariable analysis model of ECG findings by troponin elevation

	Troponin elevation		
Predictors	*Odds Ratios*	*Confidence Interval*	*p*
(Intercept)	0.23	0.01–4.67	0.336
Complete RBBB	0.95	0.59–1.50	0.815
Incomplete RBBB	1.16	0.71–1.87	0.545
Sinus tachycardia	2.07	1.42–3.03	**<0.001**
**S1-Q3-T3 pattern**	1.51	1.08–2.12	**0.016**
**ST elevation V_1**	1.00	0.63–1.56	0.989
T wave inversions V_{2-4}	1.93	1.32–2.84	**0.001**
T wave inversions II, III, aVF	1.55	1.01–2.39	**0.047**
ST depression in V_{4-6}	1.21	0.76–1.92	0.409
ST segment elevation aVR	1.51	1.00–2.25	**0.047**
SVT	1.07	0.62–1.83	0.799
Left bundle branch block with associated TWI	1.16	0.41–2.94	0.770

LVH with associated TWI	0.92	0.39–2.07	0.850
Male	1.07	0.83–1.38	0.626
African American/Black	1.54	0.84–2.93	0.170
White	1.09	0.61–2.04	0.769
Ethnicity	1.00	1.00–1.00	0.635
Age	1.02	1.01–1.03	**<0.001**
Initial heart rate	0.99	0.98–1.01	0.328
Initial shock index	2.62	1.21–5.76	**0.015**
Initial respiratory rate	1.04	1.01–1.07	**0.004**
Initial oxygen saturation on room air	0.97	0.94–1.00	**0.025**
Preceding episode of syncope	2.33	1.55–3.51	**<0.001**
Prior history of PE or DVT	1.21	0.91–1.61	0.187
No abnormal ECG pattern	0.78	0.51–1.20	0.253
Observations	1472		

R2 Tjur	0.164

* Abbreviations: RBBB = right bundle branch block, SVT = supraventricular tachycardia (including atrial fibrillation with rapid ventricular response [100 per minute]), LVH = left ventricular hypertrophy, TWI = T-wave inversion (0.5 mV negative deflection), PE = pulmonary embolism, DVT = deep vein thrombosis, ECG = electrocardiogram
