# Supplementary material for: Electrocardiographic findings associated with early clinical deterioration in acute pulmonary embolism
Source: Acad Emerg Med. 2022 Jul 20;29(10):1185–96. doi: 10.1111/acem.14554 (PMC9796434; doi:10.1111/acem.14554)
Supplement: Supplementary file 1 — Data S1 [file ACEM-29-1185-s001.zip › ACEM_14554_Table_S7_Final.pdf]

Table S7: Multivariable analysis of ECG findings by natriuretic peptide elevation

	Natriuretic peptide elevation		
Predictors	*Odds Ratios*	*Confidence Interval*	*p*
(Intercept)	0.96	0.05–21.51	0.981
Complete RBBB	1.15	0.72–1.83	0.559
Incomplete RBBB	1.21	0.76–1.90	0.420
Sinus tachycardia	1.10	0.76–1.58	0.610
**S1-Q3-T3 pattern**	1.38	0.99–1.92	0.061
**ST elevation V_1**	2.03	1.31–3.18	**0.002**
T wave inversions V2-4	2.11	1.44–3.12	**<0.001**
T wave inversions II, III, aVF	1.26	0.81–1.95	0.304
ST depression in V 4-6	0.76	0.47–1.22	0.259
ST segment elevation aVR	1.00	0.67–1.50	0.988
SVT	3.53	2.03–6.31	**<0.001**
Left bundle branch block with associated TWI	1.69	0.68–4.37	0.261
LVH with associated TWI	1.67	0.75–3.81	0.212

Male	1.35	1.07–1.70	**0.013**
African American/Black	1.68	0.97–2.95	0.069
White	0.93	0.55–1.60	0.777
Ethnicity	1.00	1.00–1.00	0.697
Age	1.03	1.02–1.04	**<0.001**
Initial heart rate	0.99	0.98–1.00	0.086
Initial shock index	3.42	1.59–7.44	**0.002**
Initial respiratory rate	1.07	1.04–1.10	**<0.001**
Initial oxygen saturation on room air	0.95	0.93–0.98	**0.001**
Preceding episode of syncope	0.80	0.52–1.21	0.294
Prior history of PE or DVT	1.21	0.93–1.58	0.153
No abnormal ECG pattern	0.85	0.58–1.23	0.383
Observations	1472		
R2 Tjur	0.182		

* Abbreviations: SVT = supraventricular tachycardia (including atrial fibrillation with rapid ventricular response [100 per minute]).
