# Supplementary material for: Electrocardiographic findings associated with early clinical deterioration in acute pulmonary embolism
Source: Acad Emerg Med. 2022 Jul 20;29(10):1185–96. doi: 10.1111/acem.14554 (PMC9796434; doi:10.1111/acem.14554)
Supplement: Supplementary file 1 — Data S1 [file ACEM-29-1185-s001.zip › ACEM_14554_Table_S9_Final.pdf]

Table S9: Full multivariable analysis model of ECG findings by computed tomography right ventricle dilatation

	**RV:LV 1.0 or greater by computed tomography**		
Predictors	*Odds Ratios*	*Confidence Interval*	*p*
(Intercept)	3.22	0.15–73.93	0.460
Complete RBBB	1.62	1.01–2.60	**0.045**
Incomplete RBBB	1.60	1.00–2.56	0.051
Sinus tachycardia	0.98	0.68–1.42	0.917
**S1-Q3-T3 pattern**	2.66	1.90–3.74	**<0.001**
**ST elevation V_1**	1.27	0.80–2.01	0.304
T wave inversions $V_{2\text{-}4}$	2.21	1.49–3.28	**<0.001**
T wave inversions II, III, aVF	0.84	0.53–1.31	0.439
ST depression in $V_{4\text{-}6}$	0.65	0.40–1.06	0.086
ST segment elevation aVR	2.88	1.91–4.38	**<0.001**
SVT	0.58	0.32–1.01	0.057
Left bundle branch block with associated TWI	0.73	0.25–1.92	0.545

LVH with associated TWI	0.60	0.25–1.40	0.252
Male	0.90	0.70–1.14	0.375
African American/Black	1.75	0.98–3.22	0.063
White	1.28	0.73–2.30	0.396
Ethnicity	1.00	1.00–1.00	0.679
Age	1.01	1.01–1.02	**0.001**
Initial heart rate	1.00	0.99–1.01	0.971
Initial shock index	4.65	2.12–10.32	**<0.001**
Initial respiratory rate	1.04	1.01–1.07	**0.007**
Initial oxygen saturation on room air	0.94	0.92–0.97	**<0.001**
Preceding episode of syncope	2.35	1.56–3.56	**<0.001**
Prior history of PE or DVT	1.18	0.90–1.56	0.226
No abnormal ECG pattern	1.07	0.72–1.59	0.739
Observations	1472		
R2 Tjur	0.187		

* Abbreviations: RV = right ventricle, LV = left ventricle, RBBB = right bundle branch block, SVT = supraventricular tachycardia (including atrial fibrillation with rapid ventricular response [100 per minute]), LVH = left ventricular hypertrophy, TWI = T-wave inversion (0.5 mV negative deflection), PE = pulmonary embolism, DVT = deep vein thrombosis, ECG = electrocardiogram
